# Supplementary material for: Non-Invasive Mapping for Effective Preoperative Guidance to Approach Highly Language-Eloquent Gliomas—A Large Scale Comparative Cohort Study Using a New Classification for Language Eloquence
Source: Cancers (Basel). 2021 Jan 8;13(2):207. doi: 10.3390/cancers13020207 (PMC7827798; doi:10.3390/cancers13020207)
Supplement: Supplementary file 1 [file cancers-13-00207-s001.pdf]

Supplementary Materials: Non-Invasive Mapping for Effective Preoperative Guidance to Approach Highly Language-Eloquent Gliomas—A Large Scale Comparative Cohort Study Using a New Classification for Language Eloquence

Sebastian Ille, Axel Schroeder, Lucia Albers, Anna Kelm, Doris Droese, Bernhard Meyer and Sandro M. Krieg

Table S1. Baseline characteristics.

		nrTMS	Awake	*p*-Value
Number of cases		100	47	
Age	mean	55.8	49.2	0.0129
	SD	15.6	14.1	
	range	20-84	25-75	
WHO I		2 (2.0)	1 (2.1)	0.0929
WHO II		12 (12.0)	8 (17.0)	
WHO III		17 (17.0)	15 (31.9)	
WHO IV		69 (69.0)	23 (48.9)	
First		63 (63.0)	24 (51.1)	0.2084
Recurrent		37 /37.0)	23 (48.9)	
Lobe	frontal	42 (42.0)	15 (31.9)	0.0016
	insular	13 (13.0)	16 (34.0)	
	temporal	21 (21.0)	2 (4.3)	
	parietal	20 (20.0)	14 (29.8)	
	right	4 (4.0)		
% of base-line pictures	mean	69.0	74.5	0.1442
	SD	20.5	17.3	
Standard picture data set		93 (93.0)	44 (93.6)	1
Alternative task		7 (7.0)	3 (6.4)	

The table shows the baseline characteristics of all included patients separated into the according groups.

Table S2. Comparison of outcome.

		nrTMS	Awake	*p*-Value
Outcome	no new	71 (71.0)	28 (59.6)	0.0642
	transient	17 (17.0)	15 (31.9)	
	permanent	3 (3.0)	3 (6.4)	
	complication	9 (9.0)	1 (2.1)	
EOR	GTR	87 (87.9)	34 (72.3)	0.0378
	STR	13 (13.1)	13 (27.7)	
Duration	mean	209.9	268.4	<0.0001
	SD	79.3	65.9	
iMRI		30/58 (51.7)	13/19 (68.4)	0.2881

The table shows the clinical and radiological outcome as well as data on the course of surgeries of all included patients separated into the according groups. Transient language deficits were defined as new surgery-related aphasia as examined five days after surgery, but the new aphasia was not persistent three months after surgery. Permanent language deficits were defined as new surgery-related aphasia as examined five days after surgery and three months after surgery. The row complication describes cases in both groups, in which the examination of the language outcome was not feasible due to a persistently decreased vigilance. The threshold for the differentiation between GTR and STR was >95% of the initial tumor volume. The percentages of iMRIs were calculated based on the period in which an iMRI was available at the department (EOR = extent of resection, GTR = gross total resection, STR = subtotal resection, iMRI = intraoperative MRI).
